# “A NEVER-ENDING CYCLE”: ANXIETY AND SLEEP AS PSYCHOSOCIAL CORRELATES OF FREEZING OF GAIT

**DOI:** 10.1093/geroni/igae098.3780

**Published:** 2024-12-31

**Authors:** Sarah Ghose, Natalie Dautovich, Claire Williams, Samantha Luehrsen, John Karlsen, GinaMari Blackwell, Leslie Cloud, Ingrid Pretzer-Aboff

**Affiliations:** Cleveland Clinic, Cleveland, Ohio, United States; Virginia Commonwealth University, Richmond, Virginia, United States; Virginia Commonwealth University, Richmond, Virginia, United States; Virginia Commonwealth University, Richmond, Virginia, United States; Virginia Commonwealth University, Richmond, Virginia, United States; Virginia Commonwealth University, Richmond, Virginia, United States; Virginia Commonwealth University, Richmond, Virginia, United States; Virginia Commonwealth University, Richmond, Virginia, United States

## Abstract

More than 50% of people diagnosed with Parkinson’s disease (PD) suffer from Freezing of gait (FoG). This debilitating symptom is characterized by an intermittent inability to initiate voluntary forward movement. Much research has focused on biological mechanisms underlying FoG, but less on psychosocial correlates. This study used a mixed-method design to elucidate associations between sleep, anxiety, and FoG. Participants were engaged in a more extensive NIH-funded parent study protocol. Thirteen people with PD (76.9% male; M age = 69 years old, SD = 6.73 years) participated. Sleep measures using actigraphy were collected one week before the parent study started. Measures of anxiety and FoG were collected pre- and post-walking through a FoG-triggering protocol. On average, participants self-reported higher State Anxiety scores at baseline and lower anxiety scores post-task. Contrarily, salivary alpha-amylase levels measuring anxiety increased steadily from baseline to post-task. Additionally, on average, participants’ self-reported (sleep diaries) better sleep across indices when compared with actigraphic measurement. Qualitatively, participants reported noticing strong associations between anxiety, sleep, and worsened FoG outcomes. Higher levels of anxiety were associated with worsened FoG. Poor sleep was associated with worsened next-day functioning and FoG. A few participants reported noticing no association between their sleep and FoG. Findings highlight the importance of anxiety and sleep for FoG outcomes, supporting prior research suggesting that higher anxiety and worse sleep may exacerbate FoG. These results suggest a need and utility for (1) further investigation and (2) the assessment and treatment of anxiety and sleep concerns beginning at PD diagnosis.

